# The Potential Role of Glycogen Synthase Kinase-3β in Neuropathy-Induced Apoptosis in Spinal Cord

**DOI:** 10.32598/bcn.11.1.1

**Published:** 2020-01-01

**Authors:** Mina Rashvand, Samira Danyali, Homa Manaheji

**Affiliations:** 1. Department of Physiology, School of Medicine, Student Research Committee, Shahid Beheshti University of Medical Sciences, Tehran, Iran.; 2. Neurophysiology Research Center, Shahid Beheshti University of Medical Sciences, Tehran, Iran.

**Keywords:** Allodynia, Hyperalgesia, Apoptosis, Neuropathic pain, GSK-3β

## Abstract

**Introduction:**

Glycogen Synthase Kinase-3β (GSK-3β) participates in several signaling pathways and plays a crucial role in neurodegenerative diseases, inflammation, and neuropathic pain. The ratio of phosphorylated GSK-3β over total GSK-3β (p-GSK-3β/t-GSK-3β) is reduced following nerve injury. Apoptosis is a hallmark of many neuronal dysfunctions in the context of neuropathic pain. Thus, this study aimed to evaluate the contribution of p-GSK-3β/t-GSK-3β ratio in spinal dorsal horn apoptosis following peripheral nerve injury.

**Methods:**

In this study, adult male Wistar rats (220–250 g) underwent Spinal Nerve Ligation (SNL) surgery. Mechanical allodynia and thermal hyperalgesia were assessed before the surgery (day 0); then, every other day up to day 8. GSK-3β selective inhibitor, AR-014418 [0.3 mg/kg, Intraperitoneal (IP)] was administrated 1 h prior to SNL on day 0, then daily up to the day 8. The GSK-3β activity and apoptosis in the lumbar section (L4, L5, or L6) of the study rat’s spinal cord were assessed by immunohistochemical and Terminal Deoxynucleotidyl Transferase dUTP Nick End Labeling (TUNEL) staining, respectively on day 8 post-SNL.

**Results:**

Following the SNL, the mechanical allodynia and thermal hyperalgesia increased on day 2 up to day 8 post-SNL. The ratio of p-GSK-3β/t-GSK-3β decreased, and the number of apoptotic cells increased in the spinal dorsal horn on day 8. However, AR-A014418 administration could increase the p-GSK-3β/t-GSK-3β ratio and decreased apoptosis in the SNL rats. In addition, AR-A014418 decreased the mechanical allodynia from day 4 up to day 8; however, it did not affect thermal hyperalgesia.

**Conclusion:**

The study findings suggested that increasing the p-GSK-3β/t-GSK-3β ratio might be a helpful strategy for reducing the apoptotic cells and subsequent neuropathic pain during peripheral nerve injury.

## Highlights

- Following the SNL, p-GSK-3β/t-GSK-3β ratio decreased in the spinal dorsal horn.
- Decreased p-GSK-3β/t-GSK-3β ratio after SNL, enhanced apoptosis in the spinal dorsal horn.
- AR-A014418 increased p-GSK-3β/t-GSK-3β ratio and decreased apoptosis and neuropathic pain.

## Plain Language Summary

Neuropathic pain is caused by damage, injury, or the dysfunction of peripheral nerves. Glycogen Synthase Kinase-3 (GSK-3) plays a crucial role in neurodegenerative diseases, inflammation, and neuropathic pain. Cell death due to apoptosis is a hallmark of neuropathic pain, but the underlying mechanisms remain unknown. So, this study attempted to evaluate the role of GSK-3β in apoptosis following peripheral nerve injury. In this study, adult male Wistar rats (220–250 g) underwent Spinal Nerve Ligation (SNL) surgery. Following the SNL surgery, the GSK-3β activity and apoptosis increased in the spinal dorsal horn, and abnormal nociceptive behavior increased. GSK-3β antagonist (ARA014418) decreased GSK-3β activity, apoptosis, and abnormal nociceptive behavior. This study suggested that the inhibition of GSK-3β might provide new insights into the treatment of neuropathic pain.

## 1. Introduction

Following Spinal Nerve Injury (SNI), the spinal dorsal horn neurons undergo distinct functional (Parker, 2017) and structural alterations (Jutzeler et al., 2016). Peripheral nerve injury results in apoptosis in the dorsal root ganglion and the dorsal horn of the spinal cord (Wiberg, Novikova, & Kingham, 2018). Apoptosis causes the loss of inhibitory systems and neuronal sensitization (Inquimbert et al., 2018). Blocking apoptosis prevents the loss of neurons and the loss of spinal GABAergic inhibition in the dorsal horn and attenuates neuropathic pain (Fu, Li, Thomas, & Yang, 2017; Scholz et al., 2005).

Glycogen Synthase Kinase 3 (GSK-3) is involved in the regulation of several processes, such as cellular function, structure, and survival (Sánchez-Cruz et al., 2018). Two isoforms of GSK-3, GSK-3α, and GSK-3β have been identified (Woodgett, 1990). The dysregulation of GSK-3 activity significantly affects apoptosis (Grimes & Jope, 2001; Jope & Johnson, 2004). The phosphorylation of GSK3β and enhanced phosphorylated GSK-3β over total GSK-3β (p-GSK-3β/t-GSK-3β) suppresses GSK3β activities and vice versa (Grimes & Jope, 2001). It has been reported that following partial Sciatic Nerve Ligation (pSNL), the ratio of p-GSK3β over the t-GSK3β expression decreases (Weng, Gao, & Maixner, 2014).

**Figure 1.**
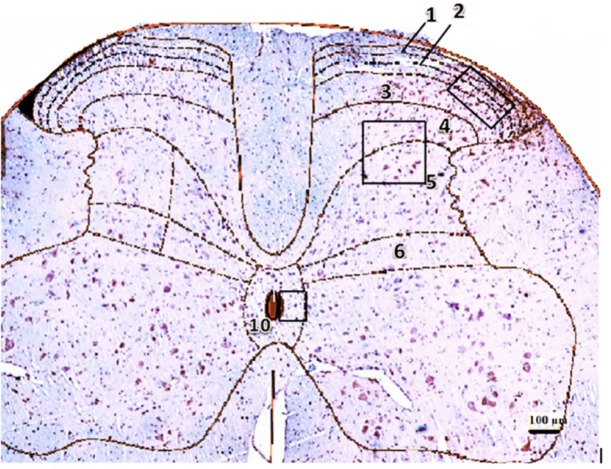
Lumbar section (L5) of the rat spinal cord Counted areas were shown in the laminae I, II, III, IV, V, and X with the dimensions of 100 μm ×200 μm, 200×200 μm^2^, and 100×100 μm^2^, respectively. Scale bar represents 100 μm.

The first report regarding the role of spinal GSK-3β in nociceptive processing was presented by Parkitna et al. (2006). They reported that the intrathecal of GSK-3β by SB216763 increased phosphorylated GSK-3β (p-GSK-3β) in the dorsal lumbar sections of the spinal cord (Figure 1) and completely inhibited the tolerance to morphine analgesia in rats (Parkitna et al., 2006). Martins et al. (2011) reported that the GSK-3β selective inhibitor ARA014418 inhibited the mechanical and cold hyperalgesia in mice’s pSNL due to its participation in descending pain control systems, like serotonergic and catecholaminergic pathways and the inhibition of proinflammatory cytokines (Martins et al., 2011).

GSK-3 plays opposite roles in extrinsic and intrinsic apoptotic pathways according to which the apoptotic signaling process is activated (Maurer, Preiss, Brauns-Schubert, Schlicher, & Charvet, 2014). Although the overexpression of GSK-3β induces apoptosis in cultured neuronal cells (Jacobs et al., 2012), there seems to be no evidence of the role of GSK-3β activity in apoptosis in the spinal dorsal horn of neuropathic rats. Thus, this study attempted to evaluate the contribution of the p-GSK-3β/t-GSK-3β ratio in neuropathy-induced apoptosis in the spinal cord and the effect of the GSK-3β selective inhibitor, ARA014418, on the protection of spinal cord neurons from apoptosis.

## 2. Methods

Adult male Wistar rats (n=36) weighing 220–250 g were obtained from Pasteur Institute, Tehran, Iran. The study animals were assigned into 4 groups (sham+vehicle, neuropathy+vehicle, sham+AR-014418, and neuropathy+AR-014418). The number of animals allocated to each group of behavioral and histological study was 6 and 3, respectively. The study animals were kept under a 2/12 h light/dark cycle, with free access to food and water. All experiments were performed according to the National Institutes of Health Guide for the Care and Use of Laboratory Animals (NIH Publication No. 80–23, revised 1996) and were approved by the Research and Ethics Committee of Shahid Beheshti University of Medical Sciences, Iran (IR.SBMU.SM.REC.1396.110). In this study, all efforts were made to minimize the number of animals used and their suffering.

In this study, neuropathic pain was induced by the SNL model, according to the method described by Chung et al. (2004), briefly, the study rats were anesthetized with an Intraperitoneal (IP) injection of 50 mg/kg pentobarbital and were placed in a prone position (Chung, Kim, & Chung, 2004). A longitudinal incision was made, and following the separation of the left paraspinal muscles from the spinous processes, the L6 transverse process was carefully removed. The left L4 and L5 spinal nerves were carefully isolated, and the L5 spinal nerve was tightly ligated using a silk thread (6-0) and transected just distal to the ligation to make sure all fibers are interrupted. The same procedure was repeated for the sham group except for the ligation and transection of the left L5 spinal nerve. After the surgery, until the end of the experiment, operated animals demonstrated no motor deficits.

The following drugs and antibodies were used in this study: AR-A014418 (N-[(4-methoxyphenyl) methyl]-N’-[(5-nitro-2-thiazolyl)] urea), anti-GSK-3β antibody (ab 93926), and anti-GSK-3β (phospho S9) antibody (ab131097). All of these drugs and antibodies were obtained from Abcam, as well as in situ cell death detection kit, purchased from SIGMA, Roche, Germany (CAS-No. 9002-93-1).

To determine the role of GSK-3β in the development of neuropathic pain and apoptosis, the GSK-3β selective inhibitor (AR-014418, 0.3 mg/kg) in a volume of 1 mL vehicle (1% DMSO in a saline solution) was IP injected into the study rats (Mazzardo-Martins, Martins, Stramosk, Cidral-Filho, & Santos, 2012). The drug or vehicle was administered one h before the sham or SNL surgery group on day 0; then, they were administrated immediately after the completion of behavioral tests daily up to day 8 (Martins et al., 2011; Weng et al., 2014). Behavioral tests, including mechanical allodynia and thermal hyperalgesia, were performed before the surgery (day 0, baseline), then every other day up to day 8 after the surgery.

To evaluate the mechanical allodynia, the Paw Withdrawal Threshold (PWT) was measured by the repeated application of a series of von Frey filaments on the left hind paw (Bahari et al., 2014). Following a 30 min acclimation period, the evoked hind paw was stimulated by a series of 7 von Frey filaments graded according to the weight (2, 4, 6, 8, 12, 15, 26, & 60 g). Each mono-filament was applied to the left hind paw for approximately 1–2 s. Each filament was applied thrice with 10 s intervals. Each trial started using 4 g filament, and if the desired response was elicited, a weaker stimulus was applied; otherwise, a stronger stimulus was applied. There was a 5 min interval between the switching of filaments (paw withdrawal response to ≥2 out of the 3 trials was considered as a positive response).

To evaluate the thermal hyperalgesia, the Paw Withdrawal Latency (PWL) following the application of noxious radiant heat to the hind paw was assessed using the plantar test apparatus (Hargreaves, Dubner, Brown, Flores, & Joris, 1988). Following a 30 min acclimation period, the PWL was measured by exposing the plantar surface of the hind paw to the radiant heat source, and the time of paw withdrawal was recorded. An automatic cut-off time (30 s) was set to minimize possible tissue damage. The PWL was measured thrice with 5 min intervals, and the average value was reported. The thermal hyperalgesia test was conducted 30 min after the completion of the mechanical allodynia test.

Immunohistochemistry (IHC) staining was used to determine the ratio of phosphorylated GSK-3β over total GSK-3β in the spinal dorsal horn. Briefly, on day 8 post-SNL or sham operation, the study rats were anesthetized with pentobarbital sodium (50 mg/kg, IP) and perfused by a solution of 4% paraformaldehyde in 0.1 M phosphate buffer saline solution (PBS, pH=7.4). The L4, L5, and L6 spinal cord segments were removed and post-fixed for 48 h at 4°C in the same fixative. Accordingly, the fixed tissues were processed by graded alcohols and xylene and were embedded in paraffin before being sectioned. In this study, 6 serial transverse sections per animal with 5 μm thickness were cut and mounted (3 sections for t-GSK-3β and 3 sections for p-GSK-3β).

The sections were deparaffinized and hydrated with 0.3% hydrogen peroxide and were further washed thrice in 0.1 M PBS; antigen retrieval was performed in 0.01 M citrate buffer. The sections were then incubated for one h at room temperature with rabbit polyclonal anti-GSK-3β (1:200, Abcam) and anti-GSK-3β phospho s9 primary antibody (1:100, Abcam) for total and phosphorylated GSK-3β, respectively.

After rinsing three times with 0.1 M PBS, the sections were exposed with mouse and rabbit specific HRP/DAB (ABC) detection kit (Abcam) for 10 min at room temperature, followed by three washes in PBS. After three rinses in PBS, they were exposed to EnVision for 20 min. Immunostained sections were counterstained with hematoxylin, dehydrated using the ascending concentrations of alcohol, followed by rinsing in xylene and coverslipped with Entellan; the stained sections were dehydrated and stabilized and mounted and viewed under an optical microscope (Olympus, Japan).

Apoptosis detection protocol is used to detect and quantify apoptosis (programmed cell death) in the spinal dorsal horn. Following perfusion, fixation, and section preparation as explained above, 3 sections per animals were deparaffinized and hydrated with two changes of 100% ethanol for 3 min and 95% ethanol for 1 min; they were then rinsed with distilled water. The sections were then exposed to proteinase K, rinsed in PBS, and treated with 3% hydrogen peroxide for 10 min to block the endogenous peroxidase activity, consequently. Moreover, these sections were subjected to the following procedures: rinsed in PBS and incubated in a mixture of enzyme and label solution for one h at room temperature; rinsed in stop wash buffer for 10 min and incubated in POD-HRP for 15 min at room temperature; rinsed in PBS and incubated with DAB chromogen for 10 min, and counterstained with hematoxylin for 1 min. After that, the sections were rinsed in the running tap water for 5 min and were dehydrated using 100% ethanol, were further treated with xylene, covered with a coverslip of Entellan, and the stained sections were viewed under the microscope (Olympus, Japan).

In the case of positive control samples, all staining steps were performed as mentioned above, except samples incubated with DNAase I, 15 min before applying hydrogen peroxide. DNAse I induced DNA strand degradation, and all of the fragmented DNA was stained brown. In the case of negative control samples, all staining steps were performed as mentioned above, except samples incubated only with the label solution instead of Terminal Deoxynucleotidyl Transferase dUTP Nick End Labeling (TUNEL) reaction mixture; no TUNEL positive cell was detected, and all of the cells turned blue because of hematoxylin application. After the immunohistochemistry or TUNEL staining, the quantification of the immunostained or TUNEL positive cells was performed as follows:

DNA damages, nuclear fragmentation leading to the formation of apoptotic bodies. The DNA fragmentation was detected via the TUNEL assay. The DNA strand breaks were detected by 3,3-diaminobenzidine (DAB) as a chromogen labeling the free 3′-OH strand. Using this procedure, apoptotic nuclei (fragmented DNA) were stained brown (positive cells). In contrast, normal cell nuclei, which have relatively insignificant numbers of DNA 3′-OH ends, basophilic cell components, like nucleic acids in the nucleus, usually turned blue by hematoxylin.

Immunohistochemistry is a method applying monoclonal antibodies to identify specific proteins in tissue sections. Detecting antigen-antibody interactions in the cells of a tissue section could be achieved by labeling the antibody with a substance that can be visualized. The 3′-Diaminobenzidine (DAB) stains the antigen-antibody complex and forms a brown precipitate at the site of antibody binding to protein (positive cells). The rest of the cells stained with hematoxylin and turned blue. In this study, we used anti-GSK-3β and anti-GSK-3β phospho S9 for labeling total and phosphorylated GSK-3β, respectively. Besides, we counted the p-GSK3B and t-GSK3β immunolabeled cells in the separate spinal sections of each group and reported the percentage of p-GSK3B/t-GSK3β immunolabeled cells in each selected area by an optical microscope. Lumbar 5 section were used for p-GSK3B/t-GSK3β ratio and apoptosis analysis. For each section, 3 fields of different spinal cord laminae (I, II, III, IV, V, and X) were randomly selected and analyzed under an optical Olympus AX70 microscopes (Japan) with a DP11 digital camera (magnification of 10×).

The number of TUNEL or GSK3B positive cells in the three field was counted, and the final count was reported as number per field, as follows: TUNEL positive cells (%) = the number of TUNEL- positive cells/total number of cells×100 The ratio of p-GSK-3β/t-GSK-3β (%)=the number of p-GSK-3β cells/total number of GSK3β cells×100. Counted areas were shown in laminae I, II, III; IV, V, and X with the dimensions of 100 μm × 200 μm, 200×200 μm^2^, and 100×100 μm^2^, respectively. Scale bar represents 100 μm.

All obtained data were presented as Mean±SEM. The behavioral data were analyzed by two-way repeated-measures Analysis of Variance (ANOVA), followed by Bonferroni posthoc test. To compare the behavioral pain tests between ipsilateral and contralateral paw, the Area Under the Curve (AUC) values in each group were measured and analyzed by one-way ANOVA, followed by Tukey’s posthoc test. One-way ANOVA, followed by Tukey’s posthoc multiple comparison tests, was also conducted in the histological studies to determine significant differences among multiple groups. The Unpaired Samples t-test was applied for ipsilateral and contralateral comparisons. Statistical analyses were performed using Graph Pad Prism (Graph Pad Prism software, USA). P<0.05 was considered statistically significant.

## 3. Results

**Figure 2.**
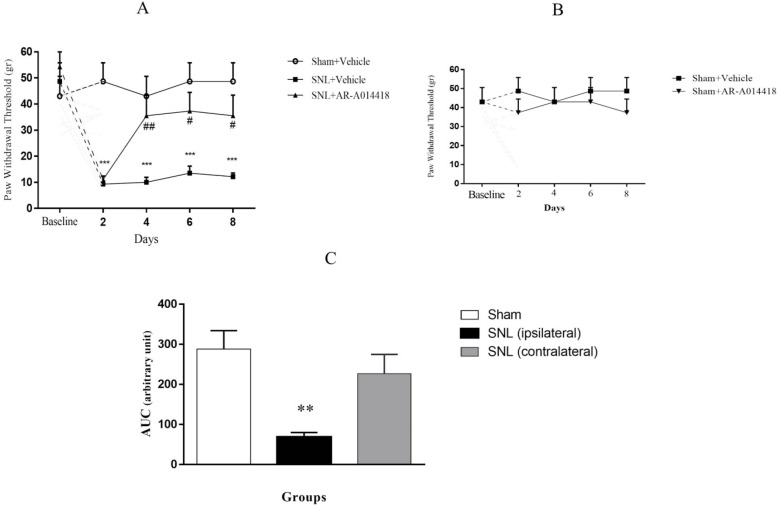
PWT in response to von Frey filaments application A. The time-course responses of PWT following von Frey filaments stimulation; ^***^P<0.001 indicates significant differences in comparison to the sham+vehicle group; ^#^P<0.05 and ^##^P<0.01 indicate significant differences compared with the SNL+vehicle group; B. AR-014418 administration had no significant effect on the sham group; C. One-way ANOVA, followed by Tukey’s posthoc test results suggested significant differences (^**^P<0.01) in the injured paw, but not in the contralateral paw of the SNL group, compared to the sham group. Data are presented as Mean±SEM; (n=6).

The two-way repeated-measures ANOVA, followed by Bonferroni posthoc test results revealed that PWT in response to von Frey filaments in the SNL+vehicle group was significantly decreased 2 days after SNL (P<0.001) and continued up to day 8 (P<0.001; Figure 2A). To determine the role of GSK-3β activity in the development of mechanical allodynia, AR-A014418 was administrated an hour prior to the surgery, then daily up to day 8. As presented in Figure 2A, the two-way ANOVA, followed by Bonferroni posthoc test analysis data, revealed that AR-014418 administration significantly increased PWT in the SNL group from day 4 to day 8 after surgery. [Treatment effect: F_(2, 10)_=15.18, P<0.001; Time effect: F_(4, 20)_=8.84, P<0.001; Treatment and time interaction effect: F_(8, 40)_=4.37, P<0.001]. Our results also suggested that AR-014418 administration has no significant effect on the sham group [Treatment effect: F_(1, 5)_=1.9, P=0.22; Time effect: F_(4, 20)_=0.08, P=0.98; Treatment and time interaction effect: F_(4, 20)_=0.25, P=0.9; (Figure 2B)].

To compare PWT between ipsilateral and contralateral paw, the AUC values in each group were measured. One-way ANOVA, followed by Tukey’s posthoc test results indicated no significant difference between the uninjured paw in SNL group and sham-operated rats (P=0.51); while the injured paw in SNL group revealed significant differences, compared to the sham-operated rats [F_(2, 15)_=8.531; P=0.003; (Figure 2C)].

**Figure 3.**
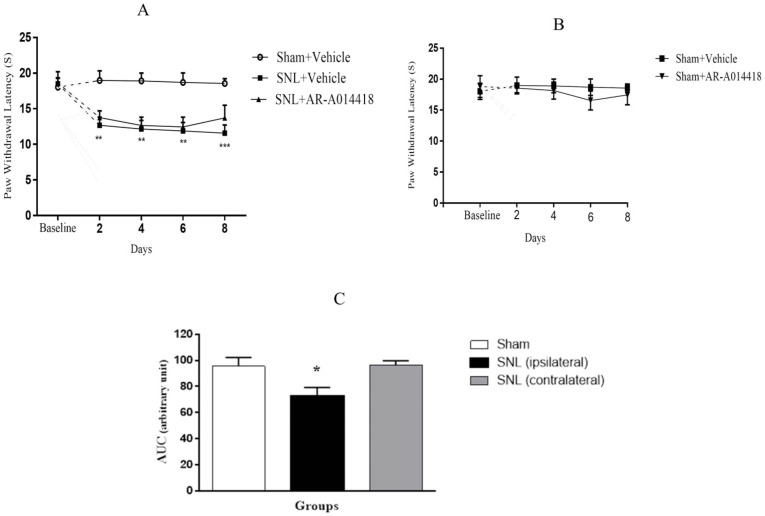
PWL in response to radiant heat stimulation A. The time-course responses of PWL following radiant heat stimulation; ^**^P<0.01 and ^***^P<0.001 indicate significant differences compared with the sham+vehicle group; AR-014418 administration had no significant effect on the SNL group; B. AR-014418 administration also had no significant effect on the sham group; C. One-way ANOVA, followed by Tukey’s posthoc test data indicated significant differences (^*^P<0.05) in the injured paw, but not in the contralateral paw of the SNL group in comparison to the sham group. Data are presented as Mean±SEM; (n=6).

As presented in (Figure 3A) PWL in response to radiant heat stimulus in the SNL+vehicle group significantly decreased on day 2 after the surgery (P<0.01; Figure 3A) and continued up to day 8 (P<0.001). Two-way ANOVA, followed by Bonferroni posthoc test data revealed no significant difference between the SNL+vehicle and SNL+AR-014418 groups after the surgery [treatment effect: F_(2, 10)_=22.7, P<0.001; time effect: F_(4, 20)_=9.16, P<0.001; treatment and time interaction effect: F_(8, 40)_=2.14, P=0.05; Figure 3A]. Our results also indicated that AR-014418 administration has no significant effect on the sham group [treatment effect: F_(1, 5)_=0.87, P=0.39; time effect: F_(4, 20)_=0.39, P=0.80; treatment and time interaction effect: F_(4, 20)_=0.20, P=0.93; Figure 3B]. Oneway ANOVA, followed by Tukey’s posthoc test results suggested no significant differences in PWL between the uninjured paw in SNL group and sham-operated rats (P=0.19); however, the injured paw in the SNL group revealed significant differences, compared to the sham-operated rats [F_(2, 15)_=5.464; P<0.05; Figure 3C]. In all of our histological studies, AR-A014418 administration revealed no significant effect on the sham-operated group; therefore, no data are presented.

**Figure 4.**
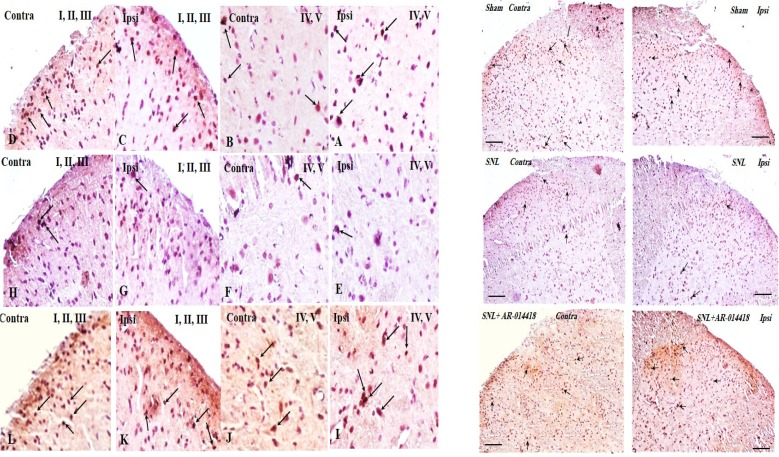
The immunohistochemistry images of p-GSK-3β in the laminae I, II, III and IV, V rat spinal cord in both ipsilateral and contralateral sides A–D: The sham+vehicle group; E–H» The SNL+vehicle group; and I–L: The SNL+AR-014418 group. Black arrows indicated some positively stained cells in the lumbar section (L4, L5 or L6) of rat spinal cord on day 8 post neuropathy or sham operation (scale bar=100 μm).

**Figure 5.**
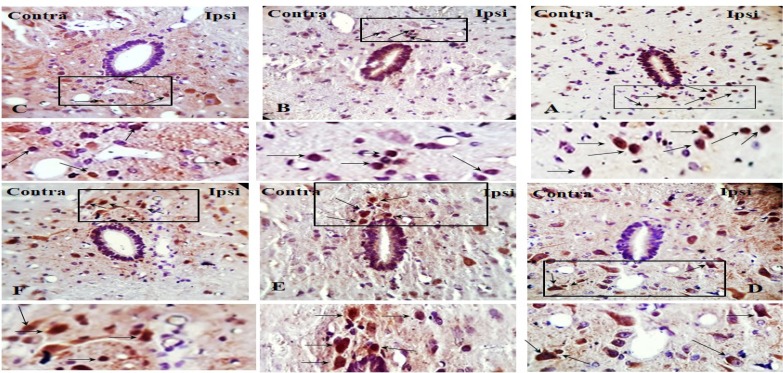
The immunohistochemistry images of p-GSK-3β in the lamina X rat spinal cord in ipsilateral and contralateral sides A. The sham+vehicle; B. The SNL+vehicle; and C. The SNL+AR-014418 groups. Black arrows indicated some positively stained cells in the lumbar section (L4, L5, or L6) of the rat spinal cord on day 8 post neuropathy or sham operation.

**Figure 6.**
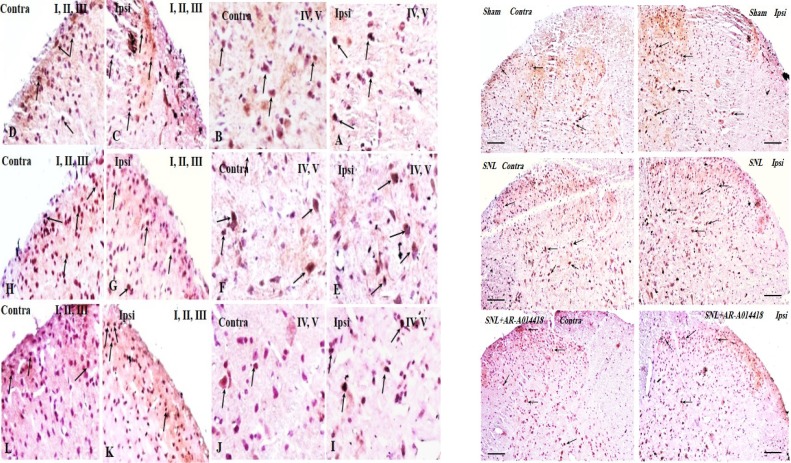
The immunohistochemistry images of t-GSK-3β in the laminae I, II, III and IV, V rat spinal cord in the ipsilateral and contralateral sides A–D: The sham+vehicle; E–H: The SNL+vehicle; and I–L: The SNL+AR-014418 groups. Black arrows indicate some positively stained cells in the lumbar section (L4, L5, or L6) of the rat spinal cord on day 8 post neuropathy or sham operation (scale bar=100 μm).

**Figure 7.**
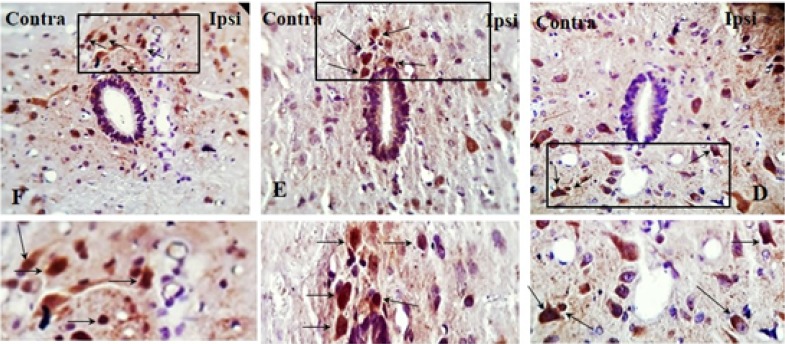
The immunohistochemistry images of t-GSK-3β in lamina X rat spinal cord in the ipsilateral and contralateral sides A. The sham+vehicle; B. SNL+vehicle; and C. SNL+AR-014418 groups. Black arrows indicate some positively stained cells in the lumbar section (L4, L5, or L6) of rat spinal cord on day 8 post neuropathy or sham operation.

We compared phosphorylated GSK-3β over total GSK-3β (p-GSK-3β/t-GSK-3β) in the spinal dorsal horn and evaluated the effect of GSK-3β inhibitor on pGSK-3β/t-GSK-3β. To understand the alteration of GSK-3β activity following SNL surgery as well as the effect of pre-emptive treatment of AR-A014418 on GSK-3β activity in the spinal dorsal horn of SNL rats, we used immunohistochemistry study for both p-GSK-3β in laminae I, I, III, IV, V (Figure 4), lamina X (Figure 5), and t-GSK-3β in laminae I, I, III, IV, V (Figure 6), lamina X (Figure 7) in the sham+vehicle, SNL+vehicle and SNL+AR-A014418 groups. For this purpose, we counted the number of phosphorylated and total GSK-3β in three distinct areas and reported the percentage of p-GSK-3β/t-GSK-3β in laminae I, II, III; IV, V, and X of the spinal dorsal horn.

The One-way ANOVA, followed by Tukey’s posthoc test data revealed a significant decrease in the p-GSK-3β/t-GSK-3β ratio in laminae I, II, III; IV, V, and X in the ipsilateral of SNL group, compared to the sham-operated rats. The pre-emptive treatment of AR-A014418 significantly increased the p-GSK-3β/t-GSK-3β ratio in the ipsilateral laminae I, II, III [F_(2, 24)_=19.23; P<0.0001]; IV, V [F_(2, 24)_=20.92; P<0.0001], and X [F_(2, 24)_=18.32; P<0.0001] of the SNL group. Furthermore, the Oneway ANOVA results revealed a significant decrease in the p-GSK-3β/t-GSK-3β ratio in laminae I, II, III, IV, V, and X in the contralateral of SNL group, compared to the sham-operated rats.

**Figure 8.**
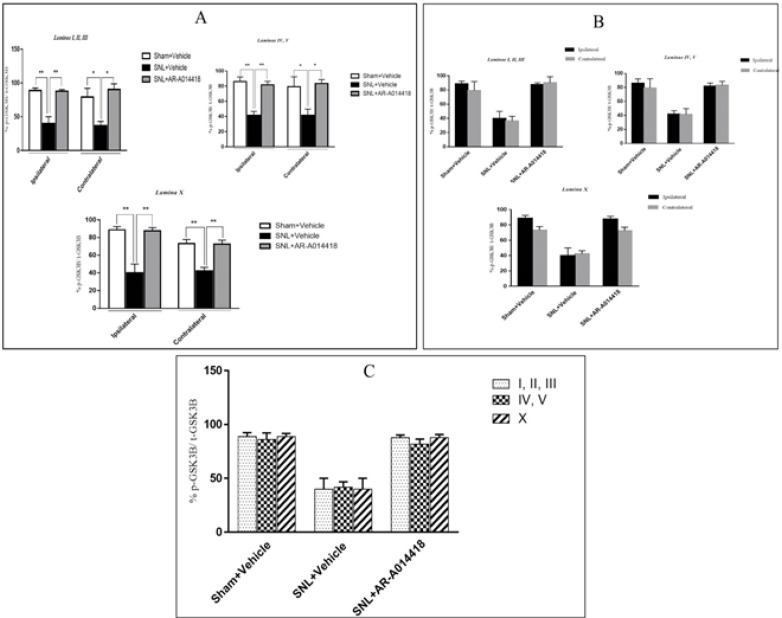
The ratio of p-GSK3β/t-GSK3β in different experimental groups A. One-way ANOVA, followed by Tukey’s posthoc test data indicated that decreased p-GSK3β/t-GSK3β ratio due to SNL surgery in laminae I, II, III, IV, V, and X of the spinal dorsal horn would be increased following the treatment of AR-A014418; ^*^P<0.05, ^**^P<0.01; B. Unpaired Samples t-test results revealed no significant difference in the p-GSK-3β/t-GSK-3β ratio between the ipsilateral and contralateral side to the injury; C. One-way ANOVA, followed by Tukey’s posthoc test, also demonstrated no significant difference among different laminae. The surface area of counted zones in the laminae I, II, III, IV, V, and X were 100 μm× 200 μm, 200× 200 μm^2^, and 100× 100 μm^2^, respectively. Data are presented as Mean±SEM.

The pre-emptive treatment of AR-A014418 significantly increased the p-GSK-3β/t-GSK-3β ratio in the contralateral laminae I, II, III [F_(2, 24)_=8.66; P=0.0015]; IV, V [ F_(2, 24)_=5.93; P=0.008], and X [F_(2, 24)_=16.22; P<0.0001] of the SNL group (Figure 8A). As presented in Figure 8B, the unpaired samples t-test data revealed no significant difference in the p-GSK-3β/t-GSK-3β ratio between the ipsilateral and contralateral side to the injury. One-way ANOVA, followed by Tukey’s posthoc test also suggested no significant difference among different laminae of sham [F_(2, 24)_=0.11; P=0.8), SNL+vehicle [F_(2, 24)_=0.01; P=0.9], and SNL+AR-A014418 [F _(2, 24)_=0.94; P=0.40; Figure 8C]. The effect of pre-emptive treatment of AR-A014418 on apoptosis in the spinal dorsal horn was investigated in this study.

**Figure 9.**
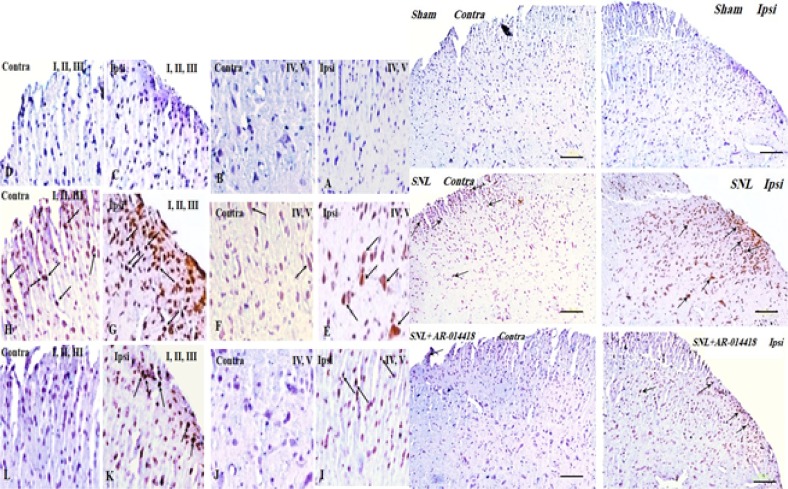
The TUNEL staining of lamiae I, II, III, and IV, V of rat spinal cord in the ipsilateral and contralateral sides A–D: The sham+vehicle group; E–H: SNL+vehicle group; and I–L: SNL+AR-014418 group. Black arrows indicate some TUNEL positive cells in the lumbar section (L4, L5, or L6) of rat spinal cord on day 8 post neuropathy or sham operation (scale bar=100 μm).

**Figure 10.**
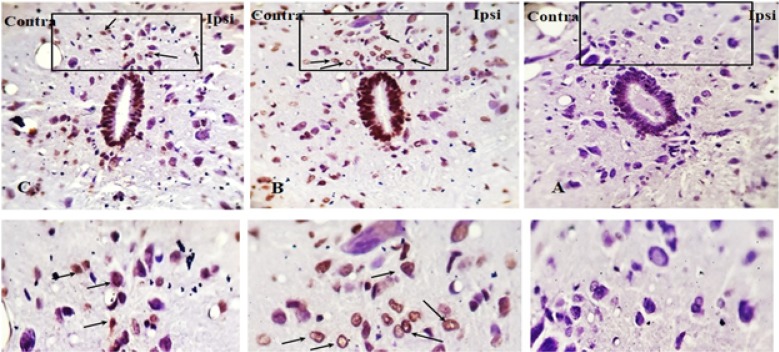
The TUNEL staining of lamia X of rat spinal cord in the ipsilateral and contralateral sides A: The sham+vehicle group; B: SNL+vehicle group; and C: SNL+AR-014418 group. Black arrows indicate some TUNEL positive cells in the lumbar section (L4, L5, or L6) of rat spinal cord on day 8 post neuropathy or sham operation.

**Figure 11.**
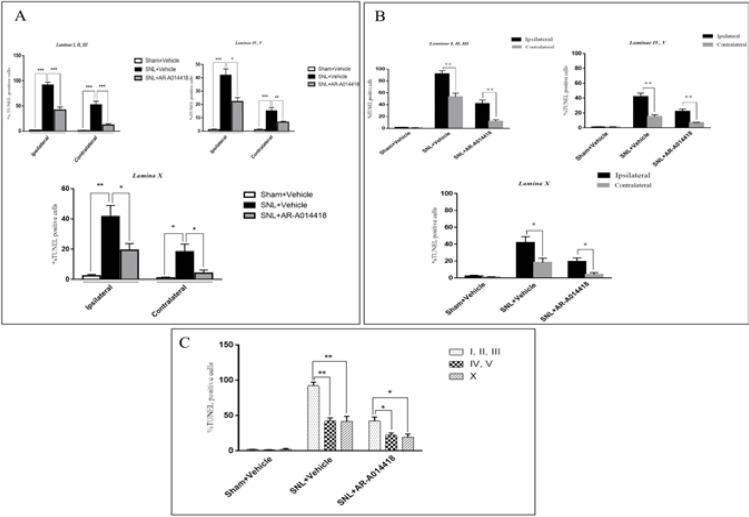
The TUNEL positive cells in different experimental groups A: One-way ANOVA, followed by Tukey’s posthoc test results, indicated an increase in the percentage of TUNEL positive cells following SNL surgery in the laminae I, II, III; IV, V, and X of the spinal dorsal horn. In addition, the treatment of AR-A014418 decreased the apoptotic cells in the SNL group; B: The Unpaired Samples t-test data revealed a significant increase in apoptotic cells in ipsilateral, compared to the contralateral side to the injury. C: One-way ANOVA, followed by Tukey’s posthoc test results also suggested a significant increase of apoptotic cells in the laminae I, II, and II, compared to the laminae IV, V, and lamina X. The surface area of counted zones in the laminae I, II, III, IV, V, and X were 100 μm^*^ 200 μm, 200^*^ 200 μm^2^ and 100^*^ 100 μm^2^, respectively. Data are presented as Mean±SEM; ^*^P<0.05; ^**^P<0.01; and ^***^P<0.001.

The TUNEL reaction labels Deoxyribonucleic Acid (DNA) strand breaks generated during apoptosis. Figure 9 and Figure 10 indicate the TUNEL staining of laminae I, II, III, IV, V, and the lamina X of lumbar section (L4, L5, or L6) of the rat spinal cord, on day 8, post neuropathy, or sham operation, respectively. As presented in Figure 11A, the one-way ANOVA, followed by Tukey’s posthoc test data revealed a significant increase in TUNEL-positive cells in the ipsilateral laminae I, II, III, IV, V, and X in the SNL group, compared to the sham-operated rats. However, the pre-emptive treatment of AR-A014418 decreased the apoptotic cells in ipsilateral laminae I, II, III [F_(2, 24)_=100.9; P<0.0001]; IV, V [F_(2, 24)_=39.59; P<0.0001], and X [F_(2, 24)_=15.98; P<0.0001] in the SNL group. The one-way ANOVA results also revealed a significant increase in the TUNEL-positive cells in contralateral laminae I, II, III, IV, V, and X in the SNL group, in comparison to the sham-operated rats. However, the pre-emptive treatment of AR-A014418 decreased the apoptotic cells in the contralateral laminae I, II, III [F_(2, 24)_=51.24; P<0.0001]; IV, V [F_(2, 24)_=22; P<0.0001], and X [F_(2, 24)_=8.22; P<0.001] in the SNL group.

The Unpaired Samples t-test data revealed that the percentage of TUNEL positive cells in the ipsilateral side was significantly higher than that of the contralateral side in the SNL and SNL+AR-A014418 groups (Figure 11B). We also observed that the percentage of TUNEL-positive cells in the laminae I, II, III was significantly higher than that in the laminae IV, V, and lamina X in the SNL+vehicle [F_(2, 24)_=24.73; P<0.0001] and SNL+ARA014418 [F_(2, 24)_=7.40; P<0.003] groups (Figure 11C).

## 4. Discussion

The present study indicated the crucial role of GSK3β in some neuronal changes, including apoptosis in the lumbar section of the spinal dorsal horn following neuropathy, for the first time. The GSK-3β is widely expressed throughout the central nervous system and is a key regulatory kinase. Our immunohistochemical study revealed that following SNL surgery, the ratio of p-GSK-3β/t-GSK3β bilaterally decreased in the laminae (I, II, III, IV, V, X) to the same extent on day 8 post-SNL. In this regard, Tang et al., (2016) argued that GSK-3β expression was markedly increased in the DRG, whereas p-GSK-3β (s9), the inactivated form of GSK-3β, decreased during nerve injury. No significant difference was observed in the expression level of GSK-3β between the ipsilateral and contralateral neurons 3–7 days after SNC. The contralateralization of pain processing in the spinal cord has been explored (Gonçalves & Dickenson, 2012). Independent of the side of the peripheral injury, we revealed that contralateral neuronal GSK3β activity is similar to that of the ipsilateral side of the injury.

Furthermore, nociceptive input is mainly concentrated in the laminae I, II, III, IV, V, and X of the spinal cord; however, little is known about the neuronal function changes under in vivo neuropathic conditions (Price, Hu, Dubner, & Gracely, 1977). The exact signaling mechanisms that cause contralateral GSK-3β activity remain unrecognized; however, studies have revealed that damage to the peripheral nerve affects the reactions of immune cells and peripheral glia (Gonçalves, Vægter, & Pallesen, 2018; Moini-Zanjani et al., 2016). These cells release several cytokines that modulate an inflammatory reaction, causing the blood-nerve barrier to become more permeable to large molecules (Olsson, 1966). Therefore, blood flow may be a nonspecific way for the delivery of factors that might activate GSK-3β in the contralateral side, like glia and proinflammatory cytokines. These factors mediate mirror-image neuropathic pain in rats (Milligan et al., 2003). We also observed that at 8 days after SNL induction, GSK3β was enhanced on both sides of the spinal cord, and the systemic injections of AR-A014418, a specific GSK3β inhibitor, reduced GSK3β activity bilaterally. Thus, peripheral nerve injury seems to produce asymmetric plasticity (Gonçalves & Dickenson, 2012).

Despite our immunohistochemical studies, our behavioral studies indicated mechanical allodynia or thermal hyperalgesia in the ipsilateral paw, but not the contralateral paw on day 8 after the surgery. Conversely, Arguis et al. (2008) reported that L5 and L6 spinal nerve ligation in rats led to mechanical and cold allodynia in the contralateral paw. A later study reported that mechanical and cold allodynia appeared in the contralateral paw on days 10 and 21, respectively (Arguis, Perez, Martínez, Ubre, & Gomar, 2008). Therefore, if our behavioral studies were continued over a longer period, we could observe the behavioral manifestation of neuropathic pain. Moreover, after neuropathy. The laterality changes over time from left to right. Both stimulus-independent and stimulus-dependent responses increased (Gonçalves & Dickenson, 2012)

According to our results, AR-A014418 (GSK-3β selective inhibitor) administration significantly increased the p-GSK-3β/t-GSK-3β ratio in the SNL group. Additionally, the daily administration of AR-A014418 significantly decreased mechanical allodynia (days 4–8 after surgery), but not thermal hyperalgesia. The thermal hyperalgesia, at least in the early stage (days 0–8), seems to be independent of GSK-3β activity. Weng et al. (2014) reported that a pre-emptive treatment of AR-A014418 in pSNL rats attenuates the development of thermal hyperalgesia in the later stage (days 8–10) of pSNL, but not in the earlier stage (days 2–4) (Weng et al., 2014).

Another study reported that mechanical allodynia and cold hyperalgesia, but not thermal hyperalgesia, decreased with AR-A014418 administration in pSNL mice (Mazzardo-Martins et al., 2012). Furthermore, mechanical allodynia and thermal hyperalgesia could be affected differently by signaling pathway molecules activated during peripheral nerve injury (Otsubo et al., 2012). Therefore, GSK-3β might have had no significant role in thermal hyperalgesia, at least within these 8 days.

The spinal nerve injury leads to apoptosis in spinal cord neurons (Martin, Reid, Verkhratsky, Magnaghi, & Faroni, 2019). Our observations revealed apoptosis in the lumbar sections (L4–L6) of the spinal dorsal horn post-SNL. The number of apoptotic cells following SNL surgery was significantly higher on the side ipsilateral to the injury compared to the contralateral side; it was also higher in the superficial laminae (I, II, III), compared to the deeper laminae (IV, V, and X). Our results are consistent with those of Whiteside and Munglani (2001), who reported a significant number of apoptotic cells in the ipsilateral dorsal horn of the spinal cord 8 and 14 days after CCI surgery. Azkue et al. (1998) reported that apoptosis in the ipsilateral laminae (I, II, III) of the spinal cord is detectable 7 days after a sciatic neurectomy. They proposed that the neurons may die as a result of deficient sensory input (Azkue, Zimmermann, Hsieh, & Herdegen, 1998). After nerve injury, neuronal discharge and ectopic activity are produced and lead to the release of excitatory neurotransmitters, including glutamate (Lazaro, North, & Dougherty, 2018); this process may result in apoptosis in the spinal dorsal horn neurons by calcium influx through the N-methyl-D-aspartate (NMDA) receptor (Inquimbert et al., 2018; Leist & Nicotera, 1998; Yamamoto, Shimoyama, & Mizuguchi, 1993).

Following this apoptosis and the removal of dorsal horn neurons, vacant synapses are generated and may induce the sprouting of Aβ fibers from deeper laminae into the superficial laminae of the dorsal horn. These sprouts would increase the sensitivity of the nociceptive system (Woolf, Shortland, & Coggeshall, 1992). More-over, this would occur if GABA-containing inhibitory interneurons die and cause disinhibition that may contribute to the establishment of chronic pain (Whiteside & Munglani, 2001; Yin, Yi, & Kim, 2018). Besides, a loss of GABAergic inhibitory tone in the neuropathic state has been reported (Sadeghi et al., 2013).

We revealed for the first time that over the activity of GSK-3β following nerve injury leads to apoptosis in the spinal dorsal horn of SNL rats, and the inhibition of GSK-3β activity by AR-A014418 decreased the apoptotic cells. This inhibitory effect of AR-A014418 on apoptosis occurred in all laminae (I, II, III, IV, V, and X) and on both sides of the spinal cord. Götschel et al. (2008) documented that the inhibition of GSK3β reduced nuclear factor κB (NF-κB) activity and attenuated TNF-α mediated apoptosis in hepatocytes (Götschel et al., 2008). Moreover, glycogen synthase kinase-3β participates in cell survival in pancreatic cancer cells (Ougolkov, Fernandez-Zapico, Savoy, Urrutia, & Billadeau, 2005). Therefore, GSK-3β plays pro- and anti-apoptotic roles in the biological systems, i.e., due to its involvement in multiple signaling pathways and phosphorylation targets.

In our study, the p-GSK-3β/t-GSK-3β ratio decreased in the ipsilateral and contralateral spinal dorsal horn 8 days post-SNL. In contrast, the number of apoptotic cells in the contralateral side was less than the ipsilateral one. However, the underlying mechanisms causing the contralateral decrease of p-GSK-3β/t-GSK-3β ratio has remained unclear; maybe the temporal nature of GSK-3β following SNL is important. For example, if we had continued the study longer, the p-GSK-3β/t-GSK-3β ratio on both sides would have been equal. Gonçalves & Dickenson (2012) argued that after left side neuropathy (SNL), the laterality changes over time from left to right (from day 2 up to day14). Both stimulus-independent and stimulus-dependent responses increased; this finding indicates that the right amygdala on day 14 is more involved than the left amygdala in the processing of sensitization of animals with neuropathy (Gonçalves & Dickenson, 2012).

Gordh and Sharma (2006) demonstrated that chronic nerve ligation impairs the spinal cord cellular microenvironment, the leakage of endogenous albumin, and the disruption of the blood-spinal cord barrier leading to the reaction of astrocytes, alteration in several neurochemical mediators, and structural changes in the contralateral side. They also declared that the structural changes were detected 2 weeks after nerve lesion and the magnitude; the intensity of neurodegenerative and structural changes in the contralateral spinal cord was less than the ipsilateral side (Gordh & Sharma, 2006). Neurons die as a result of deficient sensory input and certain structural changes, i.e., naturally time-consuming phenomenon (Azkue et al, 1998). In addition, apoptosis is secondary to GSK3β activity (Hetman, Cavanaugh, Kimelman, & Xia, 2000). AR-A014418 administration prevented apoptosis induced by SNL. Thus, if our observations were continued in a longer period, it was possible to observe the same or more apoptotic cells in the contralateral side. Accordingly, further investigations are required in this area.

## 5. Conclusion

In conclusion, this study finding suggested that reducing the p-GSK-3β/t-GSK-3β ratio could be considered as a helpful strategy for reducing apoptotic cells and subsequent neuropathic pain during peripheral nerve injury.
